# Surgical treatment for cryptoglandular and Crohn’s perianal fistulas: Protocol of an umbrella review

**DOI:** 10.1371/journal.pone.0251460

**Published:** 2021-05-13

**Authors:** Zubing Mei, Qin Feng, Peixin Du, Bin Li, Chenyang Fang, Jinghua Gu, Yue Li, Haikun Zhou, Zhuo Shao, Maojun Ge, Yazhou He, Xuejun Yang, Qingming Wang

**Affiliations:** 1 Department of Anorectal Surgery, Shuguang Hospital, Shanghai University of Traditional Chinese Medicine, Shanghai, China; 2 Anorectal Disease Institute of Shuguang Hospital, Shanghai, China; 3 Institute of Liver Diseases, Shuguang Hospital, Shanghai University of Traditional Chinese Medicine, Shanghai, China; 4 Department of Neurosurgery, Tongji Hospital, Tongji University School of Medicine, Shanghai, China; 5 Department of General Surgery, Changhai Hospital, The Second Military Medical University, Shanghai, China; 6 Department of General Surgery, Shuguang Hospital, Shanghai University of Traditional Chinese Medicine, Shanghai, China; 7 Usher Institute of Population Health Sciences, The University of Edinburgh, Edinburgh, United Kingdom; 8 Institute of Nephrology, Shuguang Hospital, Shanghai University of Traditional Chinese Medicine, Shanghai, China; Universita degli Studi di Brescia, ITALY

## Abstract

**Introduction:**

A high prevalence of cryptoglandular and Crohn’s perianal fistulas has been reported worldwide, and several surgical options are available for the management of anal fistula, with varying clinical efficacy. However, currently, the available evidence for the effectiveness of these surgical approaches are lacking and of concern in terms of the credibility and strength. The purpose of this study is to evaluate the credibility of the published systematic reviews and meta-analyses that assess the efficacy and safety of the surgical options for cryptoglandular and Crohn’s perianal fistulas through an umbrella review.

**Methods and analysis:**

A systematic search in PubMed, Embase and Cochrane library will be performed from inception to December 2020 without any language restriction. We will include systematic reviews and meta-analyses that investigate the efficacy and safety of surgical approaches in the management of cryptoglandular and Crohn’s perianal fistulas. Two reviewers will independently screen search results through reading the titles or abstracts. Relevant information will be extracted from each eligible systematic review or meta-analysis. Based on random effects model summary estimates along with their p values, 95% prediction intervals, between-study heterogeneity, small-study effects and excess significance, we will classify the evidence from convincing (class I) to weak (class IV). Findings will be summarized using quantitative synthesis combined with a narrative approach. Cryptoglandular and Crohn’s perianal fistulas will be summarized separately. Two authors will independently perform the literature search, data extraction, and quality assessment of each included systematic review and meta-analysis. Any unresolved conflicts or doubts will be resolved by discussion or by consulting a senior author. The risk of bias of the systematic reviews will be assessed using a 16-item Assessment of Multiple Systematic Reviews 2 (AMSTAR2) checklist. The strength of evidence for the included systematic reviews will be classified as "high", "moderate", "low", or "critically low" quality.

**Ethics and dissemination:**

Ethics approval is not required as we will collect data from the published systematic reviews and meta-analyses without using individual patient data. The results of this umbrella review will be published in a peer-reviewed journal and will be presented at an anorectal disease conference.

**PROSPERO registration number:**

CRD42020200754.

## Introduction

Anal fistula, also named fistula-in-ano, is a benign anorectal disease characterized by abnormal inflammatory communication between the anal canal and the perianal skin caused by infection and injury of perianal region [1]. In Europe, the estimated incidence of anal fistula is 1.2–2.8 per 10000 people, with a peak incidence between the ages of 20 and 40 years [2, 3]. In China, epidemiological studies show that anal fistula accounts for 1.67% to 3.60% of the incidence of anorectal diseases, with a ratio of male to female of 5–6: 1 [4]. More than 20% of patients with Crohn’s disease will develop anal fistula at least once in their life [5, 6], and a considerable number of patients will have recurrent attacks, so perianal fistulas are considered to be a refractory and recurrent disease, which greatly affects the quality of life of patients and places a significant financial burden to those patients [7, 8].

Cryptoglandular and Crohn’s perianal fistulas are two types of fistula with similar pathophysiology but distinct etiologies. Cryptoglandular fistulas are caused by cryptoglandular infections, which contribute to abscess formation, whereas Crohn’s perianal fistulas are mainly caused by an abnormally activated immune system, which can result in chronic inflammation and deep infection formation.

Surgery is the primary treatment option for both conditions, and several surgical techniques have been used with varying clinical efficacy in terms of recurrence rate and other postoperative complication rate reported in literatures [9–11]. In particular, the difficulty in the surgical treatment of complex anal fistula is primarily due to: (1) the difficulty of accurately locating and treating the internal openings of some types of anal fistulas; (2) the contradiction between drainage and reduction of wound size; (3) the contradiction between complete wound debridement and protection of anal function; (4) the contradiction between sphincter preservation surgery and long-term effects.

At present, the surgical approaches for patients with anal fistula include fistulotomy, seton, video-assisted anal fistula treatment (VAAFT), autologous adipose-derived stem cells (especially for Crohn’s fistula-in-ano), mucosal advancement flap, ligation of intersphincteric fistula tract (LIFT), fibrin sealant, biologic fistula plugs, synthetic fistula plug and fistula tract laser closure [12–15]. They can cure some types of anal fistula on the premise of maximum protection of anal function, but there is a lack of high-quality evidence supporting an optimal procedure for certain types of anal fistula.

Several randomized controlled trials, clinical controlled trials, comparative studies, and subsequently systematic reviews and meta-analyses, have been conducted to investigate the efficacy of surgical-related interventions on the clinical outcomes in patients with both cryptoglandular and Crohn’s perianal fistulas [16–20]. Nevertheless, the current evidence is still debatable, and high-quality evidence is required to objectively assess the clinical efficacy of certain interventions, given the rising prevalence of perianal fistulas in patients with and without Crohn’s disease. Umbrella reviews offer a similar metric and methodological framework for evaluating systematic reviews with varying populations, interventions, and outcomes. Furthermore, previously reported evidence of low quality has a significant impact on the credibility of effect estimates. The goal of this umbrella review is to systematically review all available evidence on surgical-related interventions in order to quantify their efficacy for people with cryptoglandular and Crohn’s perianal fistulas, as well as to assess the strength and credibility of the findings.

## Methods and analysis

### Protocol registration

The umbrella review will be performed according to the predesigned protocol that has been developed based on the Preferred Reporting Items for Systematic Review and Meta-Analysis Protocols (PRISMA-P) guidelines [21] (S1 Table). This project has also been registered with the International Prospective Register of Systematic Reviews (PROSPERO) database (www.crd.york.ac.uk/prospero) under the registration number CRD42020200754. The review is scheduled to be conducted between December 2020 and October 2021.

### Data sources and search strategies

We will conduct a comprehensive review of all available reviews on the topic using the methodology described in previous reports [22–24]. Umbrella reviews are defined as systematic overviews of systematic reviews and/or meta-analyses, that can be used to provide a picture of broad health-care field and assess the level of evidence for clinical practice [25].

We will conduct a systematic search of five databases, including PubMed, the Cochrane Database of Systematic Reviews, Embase, Web of Science, and Google Scholar, until December 2020. All systematic reviews, meta-analyses, and pooled analyses that investigate the efficacy and safety of surgical approaches in the management of cryptoglandular and Crohn’s perianal fistulas will be included.

Our search strategy will be comprised of the three elements listed below: (1) clinical condition (rectal fistula, anal fistula, anorectal fistula, perianal fistula and fistula-in-ano and Crohn disease); (2) interventions (LIFT, fistulectomy, fistulotomy, rectal advancement flap, anal fistula plug, fibrin glue, seton drainage and mesenchymal stromal cells); and (3) study design (systematic reviews, meta-analyses and pooled analyses), with variations for various databases (S2 Table). We will also look through systematic review registries (www.crd.york.ac.uk/prospero and www.inplasy.com). Unpublished grey literature will not be considered. We will not impose any language restrictions. A manual search of reference lists from relevant studies will be conducted.

EndNote X9 software (Thomson Reuters, Toronto, Ontario, Canada) will be used to remove duplicates and screen for literature. Two authors (ZM and QF) will independently select the potentially relevant systematic reviews by reading through their titles and abstracts. Any disagreements will be discussed with a senior author (XY) until consensus is reached. When titles and abstracts are insufficient to determine whether to include or exclude reviews, we will download full texts to determine eligibility.

Systematic reviews of randomized controlled trials and controlled clinical trials investigating the efficacy of surgery-related interventions for patients with cryptoglandular or Crohn’s perianal fistulas will be included. Cryptoglandular and Crohn’s perianal fistulas will be summarized separately. Because we only focus on surgical treatment interventions, systematic reviews of non-interventional studies (i.e., observational studies) will be excluded. Finally, only systematic reviews with quantitative data synthesis will be considered. The systematic reviews will be excluded if study-level effect estimates with 95% CIs cannot be obtained. Based on the umbrella review methodology, when multiple systematic reviews provide duplicated datasets for the same comparison, the systematic review with the greatest number of studies providing study-level effect estimates is retained for further analysis [26]. The following are the detailed inclusion criteria:

### Participants

Participants are those who have an established diagnosis of cryptoglandular or Crohn’s perianal fistulas based on accepted criteria (such as clinical manifestations and magnetic resonance imaging), without limits on age or gender.

### Interventions

Surgical interventions (vs. conventional procedures) for the treatment of cryptoglandular or Crohn’s perianal fistulas, such as ligation of intersphincteric fistula tract, fistulectomy, fistulotomy, rectal advancement flap, anal fistula plug, fibrin glue, seton drainage and mesenchymal stromal cells, will be included. Our umbrella review will primarily focus on randomized controlled trials and controlled clinical trials of the aforementioned individual interventions in the treatment of anal fistula, and the interventions of the control group involve the commonly used procedures for anal fistula that are not limited to a specific one.

### Outcome measures

Systematic reviews and meta-analyses that report one or more of the following types of outcome measures will be considered:

1. Surgical related symptoms or conditions include: surgical time, postoperative pain, postoperative complications or adverse events such as postoperative pain, bleeding, wound swelling, urinary retention, delayed healing, or healing rate.
2. Anal fistula recurrence: recurrence of anal fistula symptoms or clinical manifestations at the end of follow-up.
3. Patient’s satisfaction and functional outcomes: patient quality of life, incontinence rate, time needed for return to work or usual activities and duration of hospital stay.

### Data collection and verification

We will develop a standardized form for extracting data from each systematic review. Two authors will collect the variables listed below and cross-check the accuracy of the data. Each primary study will yield the following information: first author, year of publication, number of included studies, population involved (type of anal fistula), type of surgical interventions, outcome reported, sample size, and study-specific standardized mean differences or weighed mean differences with corresponding 95% CIs for continuous outcomes (i.e. surgical time, postoperative pain, patient quality of life, time required to return to work or usual activities and duration of hospital stay), or relative risks/hazard ratios/odd ratios with corresponding 95% CIs for categorical outcomes (i.e. postoperative complications or adverse events and recurrence of anal fistula).

### Critical appraisal

Methodological quality of the included systematic reviews will be assessed by two authors using the Assessment of Multiple Systematic Reviews 2 (AMSTAR2, an updated version of AMSTAR) tool, a 16-item checklist used to critically rate the quality of an individual systematic review as high, moderate, low and critically low based on the total score of the AMSTAR2.

### Data analysis

#### Estimation of summary effect

For each intervention and outcome measure, we will re-estimate the summary effect and the 95% CI using random effects model with the DerSimonian-Laird (inverse variance) method by combining effect estimates from previously published meta-analyses after removing duplicates. Furthermore, we will compare the direction, level of statistical significance and overlapping confidence interval for the summary effects of the association for overlapping meta-analyses that investigate the same relationships between surgical interventions and clinical outcomes in the same clinical setting, In general, we will select the most updated one for further evaluation.

#### Stratified and subgroup analyses

Stratified analyses will also be performed, with estimates summarized by disease type (cryptoglandular anal fistula vs. Crohn’s anal fistula), study design (randomized controlled trials vs. non-randomized controlled trials), total sample size (<500 vs. ≥500), follow-up period (<12 months vs ≥12 months), and the combined surgical and medical treatment for Crohn’s anal fistula (yes vs. no) across studies.

#### Heterogeneity analysis

The χ2-based Cochran’s Q test [27] and Higgins I^2^ statistic [28] will be used to test between-study heterogeneity. When the I^2^ statistic exceeds 25%, 50%, and 75%, the heterogeneity is considered low, substantial and considerable, respectively.

#### Estimation of prediction intervals

To account for between-study heterogeneity, the prediction intervals (PIs) and 95% CIs, which are used to show the expected range of true effect estimates in future studies, will also be calculated for the summary random effect estimates [29].

#### Assessment of small study effects

To investigate small study effects, Egger’s regression asymmetry test will be used [30]. In generally, smaller studies provide larger effect estimates than larger studies. In random-effects meta-analysis, a P value ≤0.10 indicates the presence of small-study effects.

#### Evaluation of excess significance

The Ioannidis’ excess significance test will be evaluated by determining whether the results of the observed number of studies (O) included in each meta-analysis (positive studies, p<0.05) differ significantly from the expected number of studies with significant results (E). To estimate the statistical power of each individual study, we will use the effect size of the largest study (smallest SE) in the included meta-analysis [31]. Excess statistical significance will be determined for each individual meta-analysis at two-sided p < 0.10 with O greater than E when the statistical significance threshold is considered.

### Grading the evidence

We will use the following criteria to evaluate the credibility of the included meta-analyses:

#### Convincing evidence (class I)

> 1000 cases, significant combined associations for random-effects calculation (p < 10^−6^), no evidence of small-study effects, no evidence of excess of significance, 95% prediction intervals excluding the null value and not large between-study heterogeneity (I^2^ < 50%);

#### Highly suggestive evidence (class II)

> 1000 cases, significant combined associations for random-effects calculation (p < 10–6), and the largest study with 95% CI excluding the null value;

#### Suggestive evidence (class III)

> 1000 cases and significant combined associations for random-effects calculation (p < 10^−3^);

#### Weak evidence (class IV)

Other associations with p < 0.05; non-significant associations: Associations with p > 0.05 [32–34].

We will conduct all statistical analyses with the STATA V.15.1 software (StataCorp, College Station, Texas, USA).

## Discussion

By incorporating evidence from published systematic reviews and meta-analyses, we will provide a comprehensive overview of the summary effects of various surgery-related interventions on the clinical efficacy of patients with cryptoglandular and Crohn’s perianal fistulas.

With the increasing prevalence of perianal fistulas of both cryptoglandular and Crohn’s disease origin, the general population bears a significant socioeconomic burden. We will assess the level of evidence for the efficacy of common anal fistula surgical techniques used worldwide, such as ligation of the intersphincteric fistula tract, fistulectomy, fistulotomy, rectal advancement flap, anal fistula plug, fibrin glue, seton drainage, and mesenchymal stromal cells.

To the best of our knowledge, this is the first comprehensive review that summarizes the efficacy of various surgical techniques for cryptoglandular and Crohn’s perianal fistulas. When sufficient high-quality data are available, we will stratify our comparisons by type of anal fistula, which will provide surgeons with high-level evidence to help them choose the best surgical technique for these patients. To account for between-study heterogeneity and provide more objective and convincing results in a future study examining the same association, 95% prediction intervals will be estimated. Furthermore, we will only include data from previously published systematic reviews and meta-analyses, which will have a higher level of evidence for this topic.

When we incorporate the high-level evidence of surgery interventions for perianal fistula from our findings, we can provide important information on clinical guidelines and therapeutic selection for those patients, such as the benefits of LIFT procedure and rectal advancement flap on the postoperative functional outcomes. Our umbrella review will have limitations as well. The included systematic reviews vary in their heterogeneity and quality. As a result, random effects meta-reanalyses will be performed for each outcome. In addition, we will use the AMSTAR 2 checklist to assess the quality of each included study.

The findings of this umbrella review will be published in a peer-reviewed journal, and we believe that the result will benefit anorectal surgeons, patients and policy-makers.

## Supporting information

S1 Table PRISMA-P checklist. (DOC)

S2 Table Search strategy for Pubmed. (DOCX)

## Abbreviations

AMSTAR2: Assessment of Multiple Systematic Reviews 2
LIFT: ligation of intersphincteric fistula tract
PRISMA-P: Preferred Reporting Items for Systematic Review and Meta-Analysis Protocols
VAAFT: video-assisted anal fistula treatment
