# Supplementary material for: Surgical treatment for cryptoglandular and Crohn’s perianal fistulas: Protocol of an umbrella review
Source: PLoS One. 2021 May 13;16(5):e0251460. doi: 10.1371/journal.pone.0251460 (PMC8118242; doi:10.1371/journal.pone.0251460)
Supplement: S2 Table — (DOCX) [file pone.0251460.s002.docx]

**S2 Table.** Search strategy for Pubmed database.

1 "Rectal Fistula"[Mesh]

2 (“rectal fistula” or “anal fistula” or“perianal fistula” or “fistula in anal” or “fistula in ano” or “anorectal fistula” or fistula-in-ano or “ Crohn disease”) [Title/Abstract]

3 1 or 2

4 (LIFT or fistulectomy or fistulotomy or “rectal advancement flap” or “anal fistula plug” or “fibrin

glue” or “seton drainage” or “mesenchymal stromal cells”) [Title/Abstract]

5 "Systematic Review" [Publication Type]

6 "Meta-Analysis" [Publication Type]

7 (meta-analysis or "meta analysis" or pooled-analysis or "pooled analysis" or "systematic review" OR "systematic revision")[Title/Abstract]

8 or/5-7

9 3 and 4 and 8
